# Single Cell Adhesion Assay Using Computer Controlled Micropipette

**DOI:** 10.1371/journal.pone.0111450

**Published:** 2014-10-24

**Authors:** Rita Salánki, Csaba Hős, Norbert Orgovan, Beatrix Péter, Noémi Sándor, Zsuzsa Bajtay, Anna Erdei, Robert Horvath, Bálint Szabó

**Affiliations:** 1 Doctoral School of Molecular- and Nanotechnologies, University of Pannonia, Veszprém, Hungary; 2 Nanobiosensorics Group, Research Centre for Natural Sciences, Institute for Technical Physics and Materials Science, Budapest, Hungary; 3 Department of Biological Physics, Eötvös University, Budapest, Hungary; 4 Department of Hydrodynamic Systems, Budapest University of Technology and Economics, Budapest, Hungary; 5 MTA-ELTE Immunology Research Group, Eötvös University, Budapest, Hungary; 6 Department of Immunology, Eötvös University, Budapest, Hungary; 7 CellSorter Company for Innovations, Budapest, Hungary; Thomas Jefferson University, United States of America

## Abstract

Cell adhesion is a fundamental phenomenon vital for all multicellular organisms. Recognition of and adhesion to specific macromolecules is a crucial task of leukocytes to initiate the immune response. To gain statistically reliable information of cell adhesion, large numbers of cells should be measured. However, direct measurement of the adhesion force of single cells is still challenging and today’s techniques typically have an extremely low throughput (5–10 cells per day). Here, we introduce a computer controlled micropipette mounted onto a normal inverted microscope for probing single cell interactions with specific macromolecules. We calculated the estimated hydrodynamic lifting force acting on target cells by the numerical simulation of the flow at the micropipette tip. The adhesion force of surface attached cells could be accurately probed by repeating the pick-up process with increasing vacuum applied in the pipette positioned above the cell under investigation. Using the introduced methodology hundreds of cells adhered to specific macromolecules were measured one by one in a relatively short period of time (∼30 min). We blocked nonspecific cell adhesion by the protein non-adhesive PLL-g-PEG polymer. We found that human primary monocytes are less adherent to fibrinogen than their *in vitro* differentiated descendants: macrophages and dendritic cells, the latter producing the highest average adhesion force. Validation of the here introduced method was achieved by the hydrostatic step-pressure micropipette manipulation technique. Additionally the result was reinforced in standard microfluidic shear stress channels. Nevertheless, automated micropipette gave higher sensitivity and less side-effect than the shear stress channel. Using our technique, the probed single cells can be easily picked up and further investigated by other techniques; a definite advantage of the computer controlled micropipette. Our experiments revealed the existence of a sub-population of strongly fibrinogen adherent cells appearing in macrophages and highly represented in dendritic cells, but not observed in monocytes.

## Introduction

Cell adhesion is a fundamental phenomenon vital for all multi and single cellular organisms. It also has an important role in developing embryos, cell-cell communication, cell migration, metastasis of tumors and inflammatory processes. Cell adhesion is mediated by cell surface receptor macromolecules, such as integrins, cadherins, selectins and members of the immunoglobulin superfamily. Cell adhesion proteins can specifically bind either the molecules of the extracellular matrix (ECM) or receptor molecules of other cells. In the direct cell-cell adhesion process cadherins play a central role mediating Ca^2+^ dependent adhesion [1]. In addition, some integrins can also form cell-cell junctions. Selectins have a lectin domain which binds to an oligosaccharide on another cell, in the presence of Ca^2+^. Members of the immunoglobulin superfamiliy mediate Ca^2+^ independent cell-cell adhesion. The main extracellular matrix receptor family is the integrin family. Integrins are assembled from two non-covalently associated subunits, called alpha and beta. Pairing of the various alpha and beta subunits yield their specific ligand affinity [1]–[3]_._

β2 integrins are leukocyte specific molecules that play an essential role in cell-cell and cell-extracellular matrix (ECM) connections. They are most abundantly expressed on neutrophil granulocytes, monocytes, macrophages, dendritic cells and NK cells. Monocytes, macrophages and dendritic cells are closely related myeloid cells, but they differ in their main function and behavior. Monocytes reside in the blood where they sample their microenvironment for invading pathogens or signs of inflammation on the endothelium. Thereby their main contact partners are pathogens, endothelial cells or ECM molecules – e.g. fibrinogen – deposited on the inflamed endothelium. Macrophages are highly phagocytic cells residing all over the body. They have powerful tools to take up and kill different microbes, apoptotic cells and other cell debris. They can migrate under different conditions but mainly reside in tissues. Macrophages make connections with the ECM, pathogens and effector T cells. Dendritic cells are the most mobile among the three cell types. They constantly search for non-self and altered self-antigens that they take up and start a migration process to transport this antigen into the lymph node to initiate different types of immune responses. This initiation process involves contact with microbes, T lymphocytes, B lymphocytes and the ECM. In cellular contacts it is very important to strengthen the specific interactions with adhesion molecules. Moreover, adhesion and cell motility is a key action in several pathologies like acute and chronic inflammation, autoimmune disorders, cancer and cardiovascular diseases [4]. The importance of β2 integrins is underlined by leukocyte adhesion deficiency (LAD) disease which is caused by a defective CD18 chain [5]. Humans with this genetic disease are unable to synthesize β2 subunits [1].

The adhesive capacity of a cell via β2 integrins depends on several factors; affinity state of the individual integrin molecules, expression level of the receptors and receptor clustering all contribute to the average affinity we measure [6]–[7]. Monocytes, macrophages and dendritic cells differ in their CD11b/CD18 (αMβ2) and CD11c/CD18 (αXβ2) expressions, monocytes bearing the least and dendritic cells expressing the most of them [8]. This basal difference combined with the fact that they are similar cell types makes them particularly suitable to study cell adherence via CD11/CD18 molecules.

Numerous techniques can be used to measure the force of cell adhesion. Most of them, including the simple washing assay [9], the spinning disk method [10] and flow chambers [11], rely on hydrodynamic shear flow removing cells from the surface [12]. However, the shear force acting on cells strongly depends on the cell shape. Although these techniques can investigate a population of cells, they do not enable single cell targeting. Furthermore, the maximum applicable shear stress is limited to measure only weak cell adhesion. Even in microfluidic channels the maximum shear stress is a few hundred Pa.

An interesting alternative to measure cellular adhesion with extremely high sensitivity is the application of evanescent field based optical biosensors [13]–[15]. Here, the biosensor signal is directly proportional to the cell-substratum contact area and also correlates with the strength of adhesion [16]. These methods can even monitor the dynamics of cellular adhesion, but are indirect and not available commercially to investigate single cells.

To directly measure the adhesion force of single cells, cytodetachment with an AFM tip [17]–[19] or micropipette aspiration [9], [20]–[22] can be applied. Both of them are inherently very low throughput methods (5–10 cells per day). Also, the temporal window on the adhesion process is narrow when using AFM due to its limited force range and technical difficulties of long term incubation of cells in the AFM. Adhesion force measurements on yeast and mammalian cells have been carried out using a modified AFM applying vacuum on cells with a fluidic micro-channel in the cantilever [23]. This technique eliminates the cumbersome AFM cantilever chemistry. Using FluidFM, a cantilever can be used for about 10 cells, which can be measured in less than half an hour, thus throughput is increased by a factor of 10 compared to conventional AFM. Additionally, the force range is enlarged up to µN allowing to widen the temporal window on the adhesion process. Optical tweezers can rather be applied for measuring subcellular forces due to their maximal strength in the pN regime [24].

To exploit the versatility of our computer controlled micropipette, we measured the adhesion force of human monocytes and their descendants: macrophages and dendritic cells on monolayers of the extracellular protein fibrinogen. We could easily perform single cell measurements on hundreds of cells in a cell culture dish.

## Methods

### Cell cultures for adhesion force measurement

#### Monocytes

Peripheral blood mononuclear cells (PBMCs) were isolated from buffy coat obtained from healthy donors and provided by the Hungarian National Blood Transfusion Service by density gradient centrifugation on Ficoll-Paque (GE Healthcare). Monocytes were isolated by negative magnetic separation using the Miltenyi Monocyte Isolation kit II (Miltenyi) according to the manufacturer’s instructions. Informed consent was provided for the use of blood samples according to the Declaration of Helsinki. Cells were cultivated in Roswell Park Memorial Institute (RPMI) medium supplemented with 10% fetal calf serum (FCS) (37°C, 5% CO_2_ atmosphere) media in Teflon coated flasks to avoid spontaneous monocyte attachment to the culture dish and were used in experiments within 24 hours [25]–[27]. Before measurements, cells were counted with a hemocytometer and 75,000 cells were placed into a 35 mm tissue culture plastic Petri dish (Greiner) coated previously with PLL-g-PEG (SuSoS) or fibrinogen (Merck). Then the cells were incubated for 30 min at 37°C, with 5% CO_2_ atmosphere. After incubation monocytes were washed several times with Hanks’ balanced salt solution (HBSS with sodium bicarbonate, without phenol red buffer, purchased from Sigma) to remove unattached cells. The experiments were carried out on freshly isolated monocytes.

#### Macrophages and dendritic cells

To generate monocyte-derived dendritic cells (MDCs), cells were cultivated in RPMI-10% FCS supplemented with 100 ng/mL rHu GM-CSF (recombinant human granulocyte/macrophage-colony-stimulating factor, R&D Systems) and 15 ng/mL rHu IL-4 (recombinant human interleukin-4, R&D Systems) for 5 days in 24 well cell culture plates (Corning) at a cell density of 5×10^5^/ml. To generate monocyte-derived macrophages (MDMs), cells were cultivated as MDCs except that only GM-CSF cytokine was added to the culture. Cytokines were supplemented every 3 days.

#### PLL-g-PEG surface coating procedure

Poly (L-lysine)-graft-poly (ethylene glycol) (PLL(20)-g{3.5}-PEG(2)) co-polymer (SuSoS), where N_Lys_ = 84 is the average number of lysine monomers in a PLL backbone, g = 3.5 is the grafting ratio (giving the number of Lys units per PEG side chain). It was dissolved in 10 mM (4-(2-hydroxyethyl)-1-piperazineethanesulfonic acid) (HEPES) buffer according to the protocol of SuSoS. 35 mm plastic tissue culture Petri dishes (Greiner) were covered with 1 ml of 1.0 mg/ml PLL-g-PEG and incubated at room temperature for 30 min. After this we rinsed the dishes with milli-Q water according to the protocol. First 10% FCS RPMI and then the suspension of cells were placed onto the coated dishes.

### Automated adhesion force measurement

#### Preparation of cell cultures for adhesion force measurement with the micropipette

Before measurements, a sterile, 9 × 9 × 5 mm (w × l × h) PDMS culture-insert containing 2 wells (Ibidi) was placed onto the surface of a 35 mm tissue culture plastic Petri dish (Ibidi). Perimeter of the wells was marked with a marker to make the border of the culture area visible when probed with the automated micropipette on the microscope. 10 µg/ml fibrinogen was put into one of the wells and it was incubated for 1 hour at 37°C in 5% CO_2_ atmosphere. After incubation, fibrinogen solution was removed and the surface was washed twice with phosphate buffered saline (PBS, Sigma). Then the insert was also removed from the Petri dish, and the entire dish was coated with 1 ml of 1 mg/ml PLL-g-PEG at room temperature according to the protocol of SuSoS. 75,000 cells were placed into the coated dish and incubated for 30 min at 37°C in 5% CO_2_ atmosphere. Then cultures were washed 3–4 times HBSS to remove floating cells.

#### Image scanning

Cells in a Petri dish were placed onto an insert (CellSorter) fitting into the 2D motorized stage (Scan IM 120 × 100 motorized stage, Märzhäuser) of the inverted fluorescent microscope (Zeiss Axio Observer A1). We chose a suitable area of the culture to be scanned using the Scanning window of the CellSorter software [28]. Phase contrast images of the culture were captured by a digital camera (Qimaging Retiga 1300 cooled CCD). During scanning in phase contrast mode, the micropipette holder arm was dislodged to let the condenser lens above the sample.

#### Cell detection

We detected cells automatically with the Local variance method of the CellSorter software in phase contrast images. Shortest path of the sorting was calculated using a travelling salesman algorithm. (See [28] for further details of image scanning and analysis.).

### Adhesion force measurements with a small diameter hydrostatic micropipette

Before measurements, a 35 mm tissue culture plastic Petri dish (Ibidi) with monocyte cells was prepared as detailed in the *Preparation of cell cultures for adhesion force measurement with the micropipette* section. Human monocytes, labeled with the fluorescent CFSE were placed onto the inverted fluorescent microscope. In these experiments, we applied a glass micropipette with an inner diameter of 5 µm instead of 70 µm to lift cells under hydrostatic conditions. The tip of the micropipette was positioned in 3-dimensions with 1 µm precision using a 40x objective lens. First we touched the surface of the Petri dish with the tip to calibrate its vertical position, then the height of the micropipette tip was adjusted to 30 µm above the surface. We positioned the tip above a cell using the joystick of the motorized stage. We adjusted the vacuum in the syringe and opened the fluidic valve. We approached the cell with the tip by moving the manipulator gently using the joystick. Distance between the tip and the bottom of the Petri dish was decreased to 10 µm. Then we lifted again the tip to 30 µm above the surface. If the cell was picked up we turned to the next cell. If the cell remained on the surface we increased the vacuum. Suction force in the [0; 0.7] µN range induced by the vacuum in the syringe was increased in steps as long as the selected cell was removed. The micropipette tended to clog after picking 5–10 cells. After clogging no more cells could be picked and we exchanged the micropipette.

#### Adhesion force measurement in microfluidic rectangular channels

As a reference measurement we used plastic flow chambers with 6 parallel channels (Ibidi, µ-Slide VI 0.1) to compare the adhesion of monocytes with *in vitro* differentiated macrophages and dendritic cells. Channels with a height of 0.1 mm were filled with 10 µg/ml fibrinogen for 1 hour, and then rinsed with PBS. Subsequently, 1 mg/ml PLL-g-PEG was introduced into the channels and incubated for 30 min to block nonspecific adhesion of cells. Control channels were treated with only PLL-g-PEG without fibrinogen coating. To achieve a cell density of 2–4 × 10^6^ cells/ml, cells were settled in a Heraeus Pico 17 centrifuge (Thermo Electron Corporation) at 300 g for 6 min. 10 µl of cell suspension was delivered into each channel. Cells were incubated in the channels for 30 min at 37°C in 5% CO_2_ atmosphere. After incubation, the flow chamber was placed onto an inverted phase contrast microscope (Olympus CKX41, 4X objective lens) to monitor the detachment of cells in the flow. A 50 ml syringe controlled by a syringe pump (New Era NE-1000) was filled with HBSS buffer, and it was connected to the µ-Slide channel via Luer. Flow rate was increased in several steps as long as most of the cells were removed from the coated surface. We applied the flow at each step for 10 s. Images were captured to determine the number of cells remaining on the surface before starting the flow and after each step. We calculated the ratio of the number of still adherent cells to the initial number of cells placed onto the surface at the beginning of the experiment.

#### Flow measurement in the micropipette

**Figure 1:**
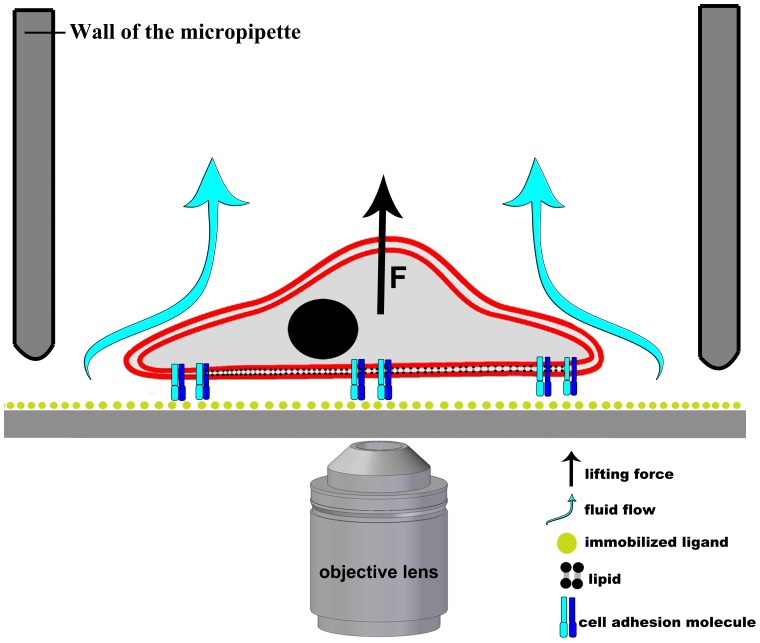
Schematic representation of the hydrodynamic adhesion force measurement on a single cell using a micropipette. Cell is shown with its nucleus and cell adhesion molecules in its plasma membrane. After coating the plastic surface with fibrinogen, we blocked nonspecific cell adhesion by the protein non-adhesive PLL-g-PEG polymer. Cells attached to the surface were scanned and recognized by software in the microscopic images captured on a motorized inverted microscope. Objective lens is shown under the cell. A glass micropipette (symbolized by its grey wall) was led to each detected cell one by one. Cell adhesion was probed by the application of a precisely controlled fluid flow through the micropipette. Experimental vacuum value measured in the syringe connected to the micropipette (Figure S1 in File S1) was converted to an estimated hydrodynamic lifting force acting on single cells according to computer simulations of the flow in the micropipette (Fig. 4).

Using our setup (Fig. 1) we positioned the tip of the micropipette to a distance of 5 or 10 µm above the surface of the 35 mm tissue culture Petri dish (Greiner) filled with 2 ml deionized water (Seralpur AP 30). PTFE tubes of the microfluidic system were partly filled with deionized water, but the end of the tube connected to a syringe was filled with air. The interface between air and water was clearly visible. The fluidic valve was then closed and the volume of the syringe was increased from an initial value of 40 ml to a higher value to generate vacuum inside. To calculate the flow rate at a given vacuum we measured the displacement of the water-air interface inside the PTFE tube during the valve opening. We opened the fluidic valve for a long duration, 20 s to minimize transient effects. The displacement of the interface in the tube was determined by a caliper. Vacuum values were calculated from the initial and final volume of the syringe and corrected by hydrostatic pressure (Hg = 270 mm) and the air content of the tube (Figure S1 in File S1). All flow measurements were repeated five times.

### Numerical simulations

We employed a Computation Fluid Dynamics (CFD) approach to compute the pressure distribution on the cell and the resulting estimated vertical lifting force. This technique solves the fundamental equations of fluid mechanics (continuity equation and equation of motion) along with the additional equations modelling the effect of turbulence. These partial differential equations are solved numerically on a so-called mesh, i.e. the fluid domain split into elementary finite volumes. The underlying mathematical formulation ensures that the conservation laws (e.g. mass conservation) are satisfied within these volumes during the solution process. The solution is obtained by iterative techniques, i.e. the solution is only approximate, however the error of the solution (i.e., the residual) is controllable: the smaller the finite volumes are (the finer the mesh is), the more accurate the solution will be but the computational resources (CPU time and memory) increase. This approach is widely used for e.g., turbomachinery [29], aerospace engineering [30]–[31], microfluidics [32]–[33] or biological flows [34]–[35], just to mention a few typical application areas. For further details we refer to [36].

The commercial CFD software ICEM CFD and ANSYS CFX 14.0 were used for meshing and fluid mechanical analysis, respectively. Due to the rotational symmetry, only 10 degrees of the geometry was built, which also allowed a radical decrease of the mesh size. The geometry is shown in Figure S2 in File S1 and the actual values are given in Table S1 in File S1. We determined the inner geometry of the glass micropipette in the digital microscopic images taken after loading the tip with a toluidine blue stain solution (Figure S3 in File S1).

Structured mesh was used to discretize the fluid domain, which made it simple to perform mesh studies. To perform grid independence studies, three meshes were used. The coarsest one consisted of 260 × 420 (x and y directions, respectively) nodes, mostly located close to the pipette-wall gap and the outlet pipe. Due to the rotational symmetry, only one mesh cell was used in the circumferential direction. The next two meshes were obtained by doubling the previous mesh number, resulting in a total number of 109 k (coarsest mesh), 438 k (regular mesh) and 1.747M (finest mesh) cells. As the culture medium is similar to water in terms of its density and viscosity, we ran the simulations with the parameters of water (ρ = 998 kg/m^3^, ν = 10^−6^ m^2^/s).

#### 3D simulations

To analyze the effect of cell shape and positioning offset, we ran additional 3D simulations (Figure S4 in File S1). These computations resolved the 3D surroundings of the cell and the pipette fully, but due to the lost axial symmetry (in case of positioning offset), a courser mesh had to be used to reach reasonable computational time. 3D mesh consisting of 547 k cells provided similar results to the simulations run in the 10 degrees sector of space described above. Minute (<10%) deviation is attributed to the coarser mesh in 3D. Computations were run with a hemisphere model of the cell with a radius of *R* in the center (axis of the micropipette) and also with the same model cell on the surface but pushed out of the center by 5 µm to estimate the impact of the error of micropipette positioning on the lifting force. To analyze the effect of cell shape on the lifting force we used an oblate hemispheroid model cell with a major radius of *R* but minor radius (height) of *R/2, i.e.,* a flatter cell. All 3D computations were run at 10 µm pipette height.

#### Boundary conditions

The boundary condition on the OP surfaces (Figure S2 in File S1) was set to opening, with 100,000 Pa average absolute pressure, which allows both in- and outflow without specifying the direction of the flow through the surface. The side walls were set to symmetry, which does not allow the formation of radial velocity components. Both the surface of the cell under the micropipette and the wall of the micropipette were modelled by no-slip boundary condition (NSW in Figure S2 in File S1). The upper end of the pipette (where the fluid leaves the domain) was set to prescribed flow rate, allowing the formation of the outlet velocity profile. The bottom of the Petri dish required special attention when defining the boundary conditions. The majority of the pressure drop (friction losses) is generated on this surface and it is known that the classic no-slip wall boundary condition does not necessarily hold in the case of micro-scale fluid mechanical applications with partially hydrophobic surfaces [37]–[43]. However, determining the so-called “slipping depth” – the virtual depth where the velocity profile reaches the zero value - is rather cumbersome as one needs to measure the velocity profile in the vicinity of the wall. Moreover, the wall treatment of ANSYS CFX allows (besides the standard free-slip and no-slip boundary condition) only to specify the shear stress at the wall, i.e. the slope of the wall-parallel velocity component in the wall-normal direction. Hence we decided to perform each computation twice: with no-slip and free-slip settings at the bottom of the Petri dish and compare the results with experimental measurements of the flow rate. The real-life velocity profile (and other integral quantities such as the pressure drop) is expected to lie between these two extreme cases. As the results of simulations with the free-slip condition on the bottom of the Petri dish was very close to the experimental calibration curve, we calculated the lifting force acting on the cell from these simulations.

#### Turbulence models and convergence

Three turbulence models were tested: (a) laminar (no turbulence model), (b) k-ε and (c) SST. See [44] for details. The governing equations (continuity equation, equation of motion and turbulence models) were discretized with second-order spatial scheme; the time scale was set to the “auto” option (CFX uses damped unsteady solver for solving steady problems. Computations were run up to the point where the RMS of the residuals (local errors) fell beneath 10^−5^ and the global imbalance was less than 0.1%.

For validation purposes, the first set of computations did not include the model cell under the micropipette. A series of computations were run with flow rates from 2 to 20 µl/s, using every combinations of the three meshes, the turbulence models and free slip/no slip boundary conditions at the bottom of the Petri dish (i.e., 36 runs). These tests showed that the finest grid is needed: the integral quantities, notably the pressure drop changed significantly between the course and the regular mesh and also varied more than 5% when using the finest grid, compared to the regular grid. They also revealed that the flow becomes turbulent at higher flow rates, which suggested the use of the SST turbulence model.

### Statistical analysis

All adhesion data shown in the figures were analyzed by the two-sample unpaired (one-tailed) t-test for comparing samples with 95% confidence.

### Ethics Statement

Blood samples were taken from healthy blood donors after written consent with ethical permission of the Scientific Research Ethics Committee of the Medical Scientific Board of Ministry of Human Resources (ETT TUKEB 55627/2012/EKU 837/PI/2012). The Scientific Research Ethics Committee of the Ministry specifically approved these studies.

## Results

### Single cell adhesion force measurement using the automated micropipette with an inner diameter of 70 µm

After coating the plastic surface of the Petri dish with fibrinogen, we blocked nonspecific cell adhesion by the protein non-adhesive PLL-g-PEG polymer [45]–[48]. Cells attached to the surface were scanned and recognized by software in mosaics of microscopic images captured on a motorized inverted microscope. A glass micropipette with an inner diameter of 70 µm attached to a vertically motorized micromanipulator was maneuvered to each detected cell one by one [28], [49]. Cell adhesion was probed by the application of a precisely controlled fluid flow through the micropipette (Fig. 1). The adhesion force of cells could be accurately measured by repeating the pick-up process with increasing vacuum.

#### Micropipette in action

After cell detection the microscope pillar with the condenser lens was tilted backwards and the micropipette (BioMedical Instruments, Germany) in its holder arm was rotated above the sample [49]. Using the joystick of the vertically motorized micromanipulator (Micromanipulator HS6/3, Märzhäuser), the micropipette was carefully driven to approach and touch the bottom of the Petri dish in order to precisely calibrate its vertical position. The positioning accuracy of the micropipette was ∼1 µm. The micropipette was illuminated by a white LED making its tip visible in the microscope. During the experiments, the micropipette was moved up-and-down by the micromanipulator. We ran experiments with two different height values of the micropipette (5 and 10 µm) to show that the method can be applied stably, not only at one specific set of parameters. Approximately half of the experiments were carried out with 5 and the other half with 10 µm. Before picking up the first cell, culture medium was let into the micropipette to avoid the osmotic shock of cells. Cells or cell debris practically never clogged the large, 70 um aperture. To avoid picking up more than one cell per measurement, we excluded cells from the experiment when having neighbors in the close proximity [28].

Vacuum in the range of [0,22] kPa was generated in the syringe using a syringe pump. After positioning the micropipette above a cell, the valve was opened for 20 ms (Figure S1 in File S1). Height of the micropipette above the Petri dish was adjusted to 5 or 10 µm. After each cycle of the adhesion force measurement, the region of interest (ROI) of the Petri dish was scanned again and the vacuum was increased to the next level. The micropipette visited again each location determined according to the initial scanning. Suction force was increased until most of the cells were removed. (Pick up parameters: Valve1∶20 ms, Valve2∶0 ms, Delay: 0 ms).

#### Ratio of adherent cells

We counted the number of cells in the images before and after each cycle of the adhesion force measurement, and calculated the ratio of still adherent cells to the cell number placed onto the surface at the beginning of the experiment.

#### Cell adhesion and the hydrodynamic lifting force acting on single cells

**Figure 2:**
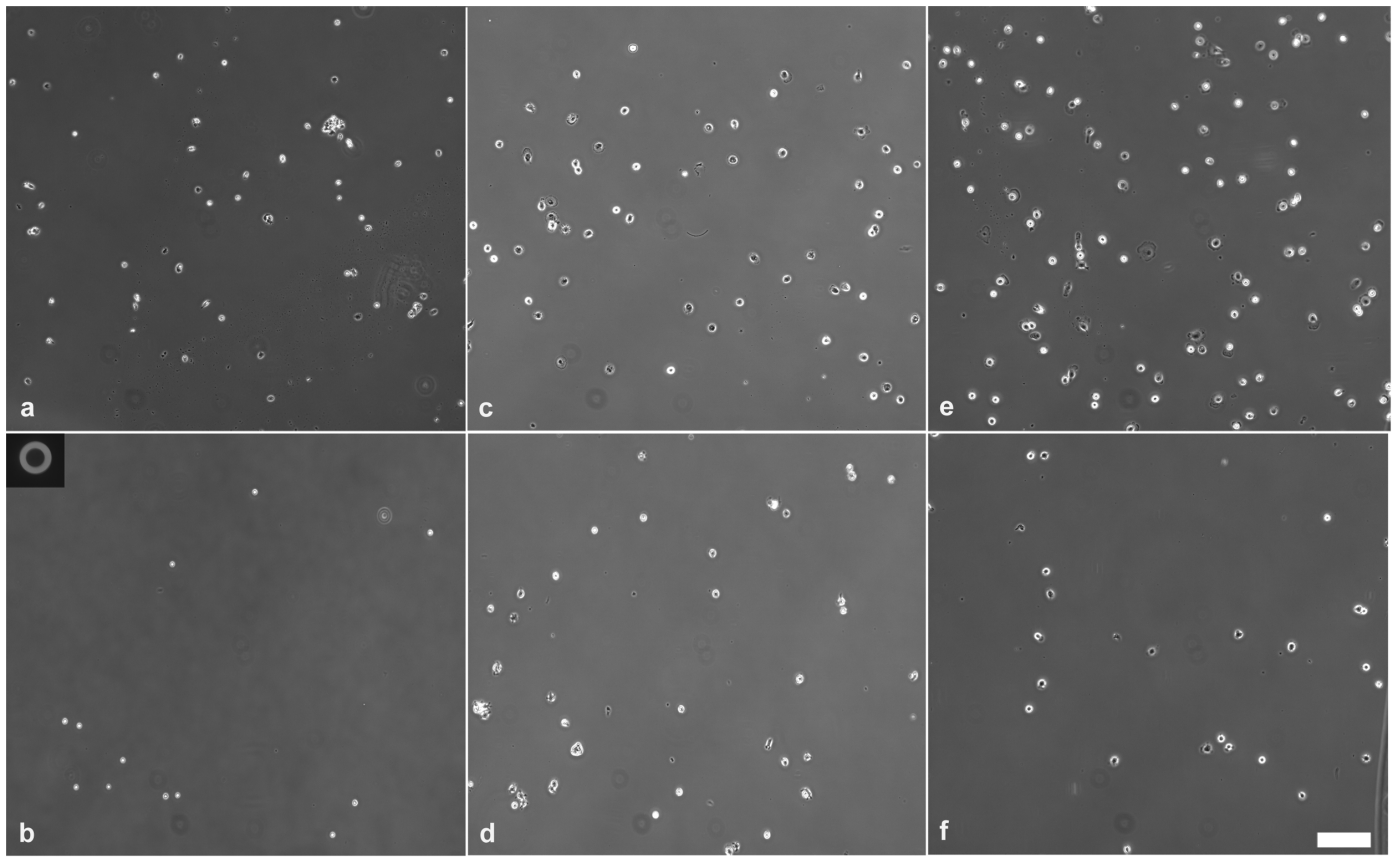
Images of adherent monocytes (*a, b*), and those of their *in vitro* differentiated descendants: macrophages (*c, d*) and dendritic cells (*e, f*) on the fibrinogen coated and PLL-g-PEG blocked surface (*a, c, e*), and on the control surface without fibrinogen coating but also blocked by PLL-g-PEG (*b, d, f*) before applying vacuum by the automated micropipette. Region of interest (ROI) of the Petri dish was scanned by the motorized microscope. Cells were detected automatically. After we adjusted the vacuum in the syringe, the micropipette visited and tried to pick up the detected cells one by one. After each cycle of the adhesion force measurement, the ROI of the Petri dish was scanned again and the vacuum was increased to the next level. The micropipette visited again each location determined according to the initial scanning. In the upper left corner of panel (*b*) we show the aperture of the glass micropipette with an inner diameter of 70 µm. Scale bar: 100 µm.

**Figure 3:**
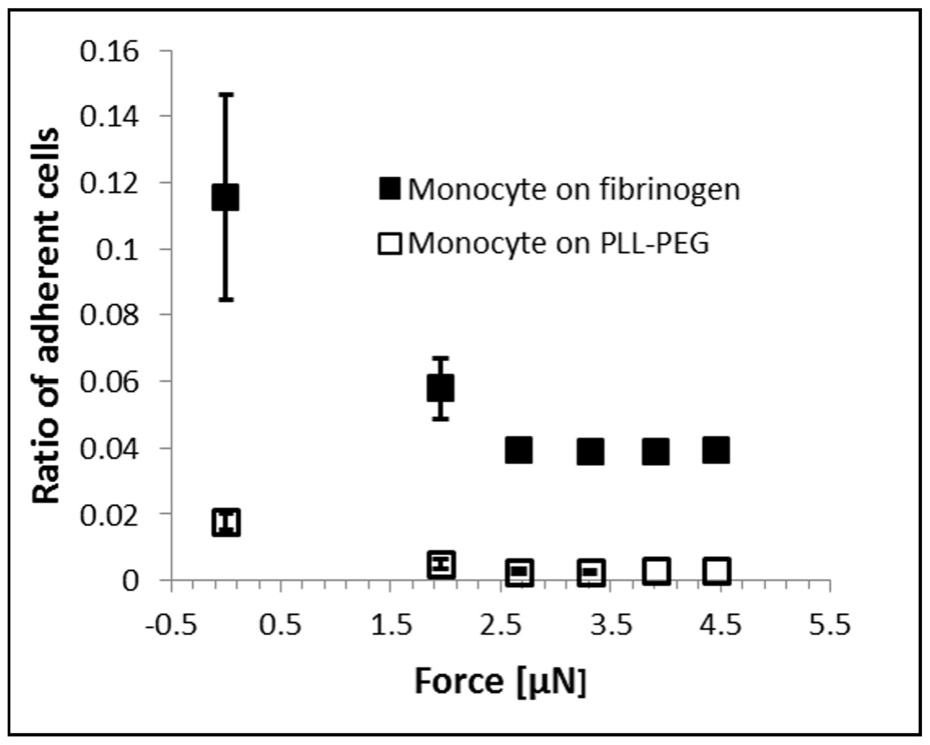
Ratio of adherent monocytes on fibrinogen and PLL-g-PEG surfaces at different lifting forces, as was measured by the automated micropipette. Experimental vacuum value in the syringe was converted to an estimated hydrodynamic lifting force acting on single cells according to computer simulations of the flow in the micropipette (Fig. 4).

**Figure 4:**
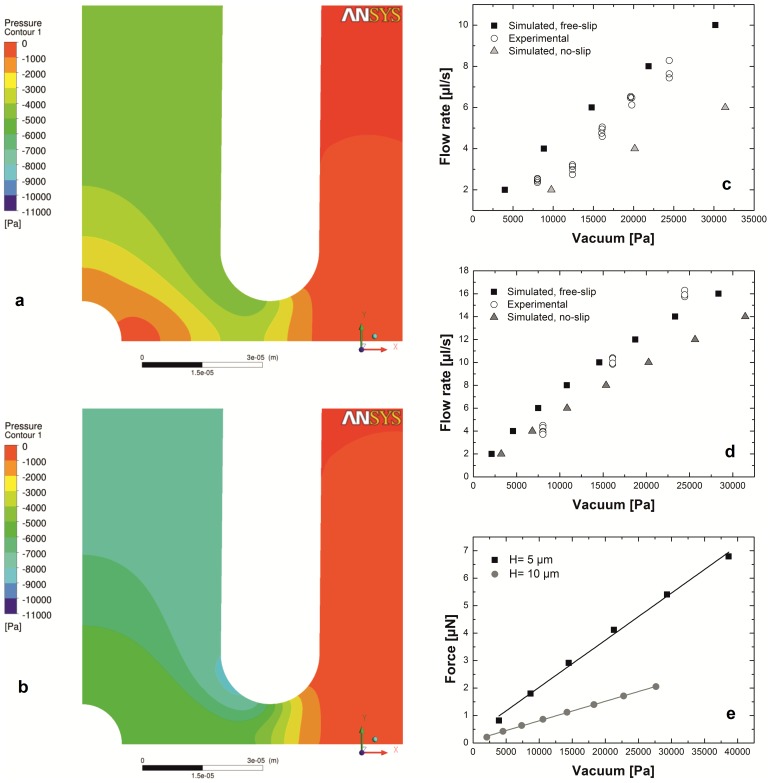
Results of the numerical simulations. Typical pressure distribution in case of free-slip (a) and no-slip (b) boundary conditions imposed on the bottom of the Petri dish. The flow field has an axial symmetry, and only the right half of the geometry is shown in the side views. The real-life velocity profile (and other integral quantities such as the pressure drop) is expected to lie between these two extreme cases. Distance between the tip of the micropipette and the bottom of the Petri dish: H = 10 µm. Flow rate: 6 µl/s. To validate the results of simulations we compared the simulated flow rate of the micropipette to the experimental values as a function of the vacuum value with H = 5 µm (c) and H = 10 µm (d) taking into consideration corrections due to gravity, pressure drop in the PTFE tube and the flow velocity in the micropipette (Figure S1 in File S1). Simulation with free-slip condition on the bottom of the Petri dish proved to be a better approximation of the experiments than the no-slip simulations. Thus we determined the lifting force (e) acting on the hemisphere model of the cell on the basis of the free-slip simulations as a function of the vacuum applied to the micropipette. With a linear fitting we found the following relation between the hydrodynamic lifting force (F_L_) and the vacuum (V) applied to the micropipette: F_L_ = 0.172 [nN/Pa] * V +311 [nN] (R^2^ = 0.996) if H = 5 µm. F_L_ = 0.071 [nN/Pa] * V +961 [nN] (R^2^ = 0.999) if H = 10 µm. We used these coefficients to convert the experimental vacuum values to an estimated lifting force.

We investigated the adhesion of human primary monocytes and monocyte-derived *in vitro* differentiated macrophages and dendritic cells to fibrinogen (Fig. 2). We measured all cell types from three different human donors. Total number of cells probed by the micropipette was n = 177 for monocytes, n = 588 for macrophages and n = 522 for dendritic cells. We coated the Petri dish with fibrinogen, then with the PLL-g-PEG polymer. PLL-g-PEG coating without fibrinogen was used as a control surface. First, we investigated the adhesion force of monocytes in the [0; 4.5] µN range (Fig. 3) using the automated micropipette. The adhesion force of most cells fell into the [0; 2] µN interval, and therefore monitoring of differentiated cells was continued in this narrower range. Experimental vacuum value in the syringe was converted to hydrodynamic lifting force acting on single cells according to computer simulations of the flow in the micropipette (Fig. 4). To estimate the lifting force acting on a real cell (Fig. 1), the total force acting on a model cell (hemisphere with a diameter of 20 µm, Figure S2 in File S1) was determined in simulations while considering both the pressure and the shear stress distribution on the cell surface. (Shear stress was negligible as compared to the negative pressure on the surface of the hemisphere.) The correlation in Fig. 4e is close to linear. Slope of the curve depends on experimental parameters, mainly the aperture of the micropipette and the distance of the micropipette from the surface. Linear correlation implies that under similar experimental conditions the adjusted vacuum value will be proportional to the lifting force, i.e., computer simulations are not necessary to gain comparative results.

We found that most cells were picked up with an estimated lifting force of 2 µN or lower. However, adhesion force of monocytes showed a wide distribution (Fig. 3) starting with a negative slope in the interval of [0; 2.5] µN. The remaining 4% of the cells with high adherence could not be removed even when applying a maximum force of 4.5 µN. Similar negative slope in the low adhesion regime has been observed earlier, when platelets adhered to fibrinogen were measured in a flow chamber [50].

**Figure 5:**
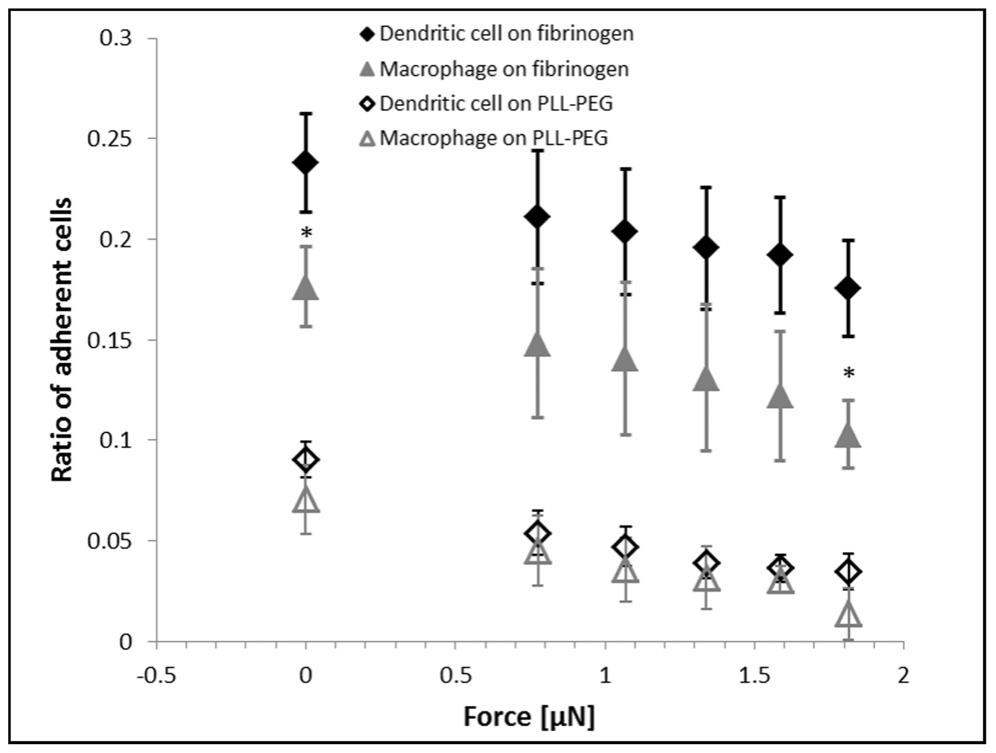
Ratio of adherent dendritic cells and macrophages on fibrinogen and PLL-g-PEG surfaces at different estimated lifting forces, as was measured with the automated micropipette. * indicates significant difference between the ratio of dendritic cells and macrophages on fibrinogen, P<0.05 (t-test).

On the basis of the results achieved with monocytes, we studied the adhesion of macrophages and dendritic cells in the [0,2] µN regime of the adhesion force (Fig. 5). Significantly more macrophages and dendritic cells stayed attached at 0–2 µN than monocytes. Dendritic cells and macrophages behaved similarly, but the mean ratio of adherent dendritic cells was higher at all vacuum levels. In only two cases, however, the difference between the adherence of macrophages and dendritic cells was significant: at zero and maximum lifting forces. On the PLL-g-PEG surface dendritic cells showed slightly higher adhesion than macrophages.

#### Effect of cell shape and positioning offset on the hydrodynamic lifting force

To analyze the effect of cell shape and positioning offset we ran 3D simulations (Figure S4 in File S1). Positioning offset had negligible effect on the hydrodynamic lifting force. We used an oblate hemispheroid model cell with a major radius of *R* but minor radius (height) of *R/2, i.e.,* a twice as flat cell to investigate the effect of cell shape. The effect of cell shape was significant only in case of the free-slip boundary condition at the bottom of the Petri dish.

### Cell adhesion measured in microfluidic channels

**Figure 6:**
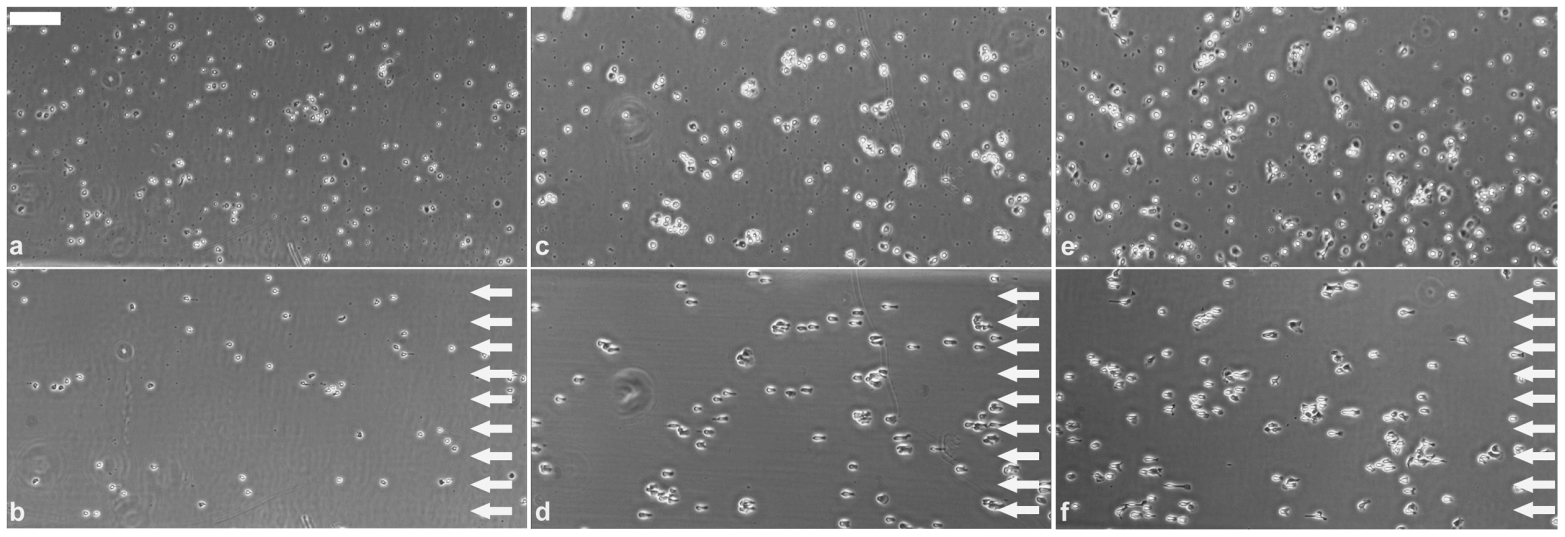
Images of adherent monocytes (a, b), and those of macrophages (c, d), and dendritic cells (e, f) on fibrinogen coating in the microfluidic channel of the flow chamber. Arrows indicate the direction of flow (b, d, f). Flow could easily remove monocytes. Macrophages (d) and dendritic cells (f) remained on the surface but became elongated at high shear stress. When the flow rate was further increased most cells detached from the surface. To give an insight into the morphology change of cells, figure shows images captured at the following shear stress values: 0 Pa (a, c, e); 21.3 Pa (b); 128.1 Pa (d), and 181.4 Pa (f). In the experiments we used the same sequence of shear stress values for all three cell types. Scale bar: 100 µm.

**Figure 7:**
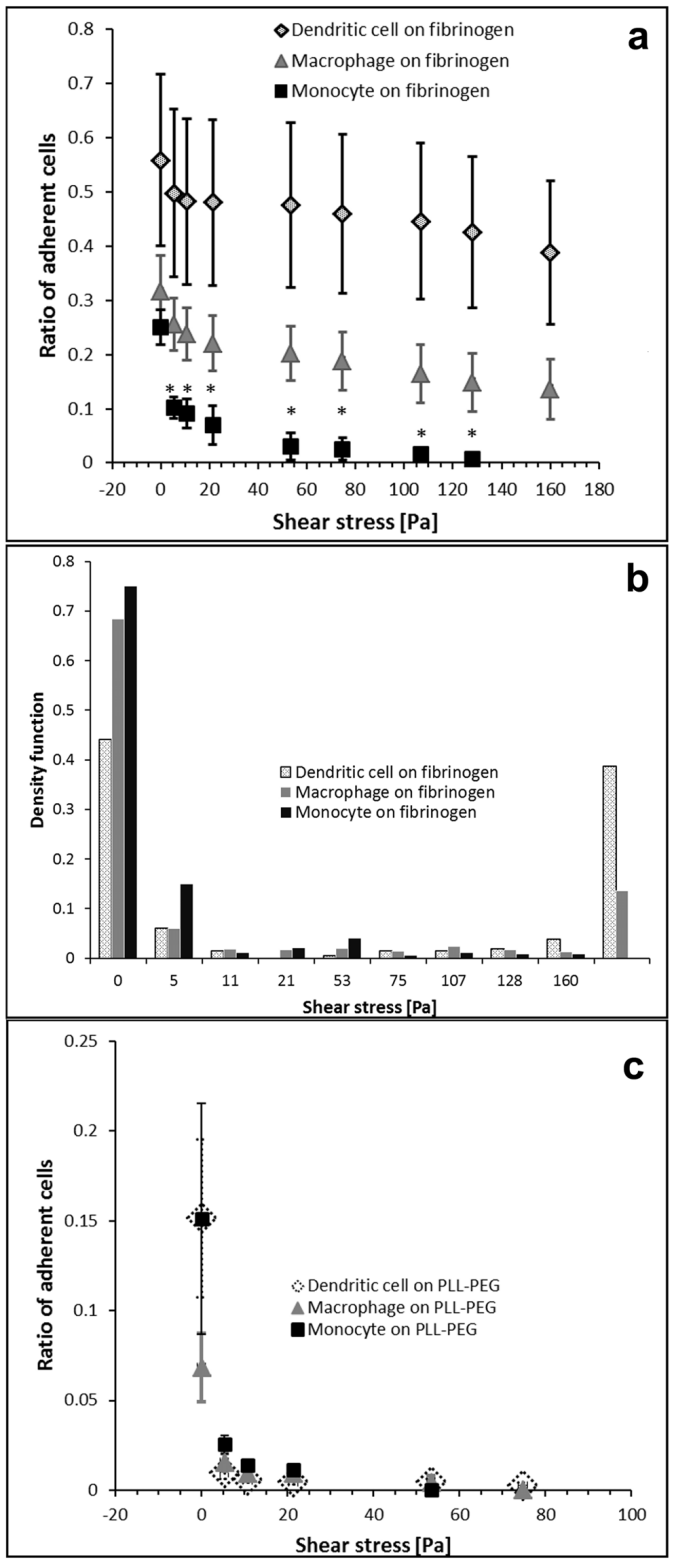
Result of the shear stress measurements. (a): Ratio of adherent dendritic cells, macrophages and monocytes as a function of the shear stress applied in the fibrinogen coated and PLL-g-PEG blocked microfluidic flow chambers. * indicates significant difference between the ratio of adherent monocytes and that of the differentiated cells on fibrinogen, P<0.05 (t-test). Difference between macrophages and dendritic cells was not statistically significant in this experiment. (b): Same results as shown in panel (a) but presented as the density function of the distribution of cells. Instead of the shift of the distribution measured with monocytes a new peak appears in case of macrophages and dendritic cells at high adhesion strength. (c): Cell adhesion to the PLL-g-PEG coated surfaces of the microfluidic channel measured and presented similarly to (a). Data of the three cell types collapse to a single curve on this weakly adherent surface. Most cells are washed away with a very low shear stress.

The microfluidic channels of the flow chamber were coated similarly to the Petri dish used in the micropipette experiments (Fig. 6). We observed a marked effect of the flow on cell morphology in case of dendritic cells and macrophages. They became elongated and aligned to the direction of the flow. Although this phenomenon is well-known in case of endothelial cells [51] it is less documented for leukocytes. Adhesion of monocytes was significantly lower than that of the differentiated cells at all shear stress values except the zero stress (Fig. 7 *a*). Difference between macrophages and dendritic cells was not statistically significant in this experiment. The mild but consequently observed difference between these two cell types clearly lies in the amplitude of peaks at very low and very high adhesion strengths (Fig. 7 *b*). New peak appearing in case of macrophages and dendritic cells at high adhesion strength reveals the existence of a sub-population of highly adherent cells among macrophages and dendritic cells, which is missing from monocytes. Data of the three cell types collapse to a single curve on the weakly adherent PLL-g-PEG surface. Most cells could be washed away with a very low shear stress from PLL-g-PEG (Fig. 7 *c*).

In summary, similar results were obtained with both the microfluidic shear stress channel and the automated micropipette, but only the latter technique was able to reveal a significant difference between macrophages and dendritic cells (Fig. 5) at the lowest and highest forces. The shear force needed to remove cells in the microfluidic channel was 2 orders of magnitude lower than the hydrodynamic lifting force measured by the micropipette.

### Adhesion force measurements with a small diameter hydrostatic micropipette

**Figure 8:**
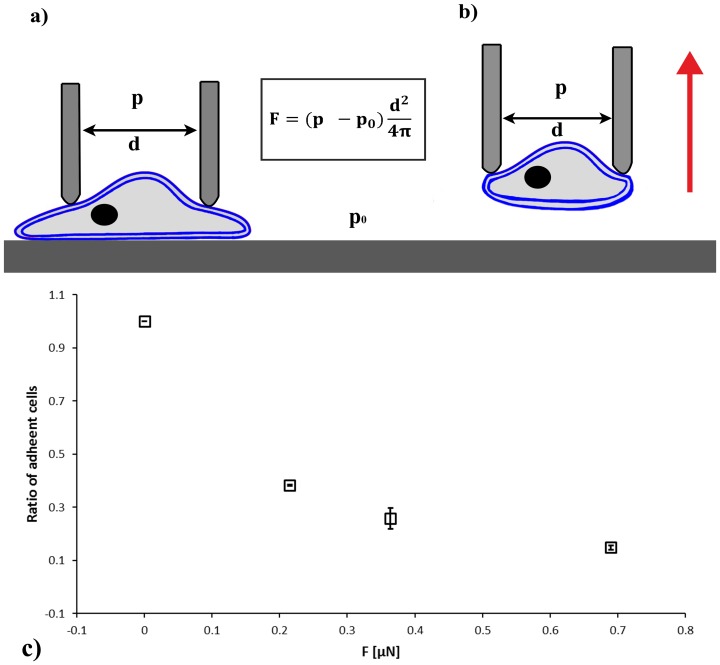
Schematic illustration of adhesion force measurements on individual monocyte cells using the step-pressure micropipette manipulation technique. a) The tip of the micropipette with an aperture of 5 µm was positioned above the selected cell with a diameter of ∼15 µm cell attached onto the fibrinogen coated surface. We positioned the tip above a cell, adjusted the vacuum in the syringe, and opened the fluidic valve constantly. We approached onto the cell with the tip gently until we touched it. Then we lifted again the tip to 30 µm above the surface. Red arrow indicates the motion of the micropipette when detaching the cell from the surface. If the cell was picked up we turned to the next cell. If the cell remained on the surface we increased the vacuum. Suction force was increased in steps as long as the selected cell were removed. We calculated the ratio of adherent cells remaining on the surface after applying the next step of vacuum (panel c). We normalized the number of adherent cells by the total number of cells probed in the experiment. Number of cells washed away from the surface before the measurement was not considered here to decrease standard error according to the consensus, when the number of probed cells is low, e.g., in AFM experiments. Data need to be rescaled to compare to Fig. 3, i.e., normalized by the ratio of initially adherent cells. Step-pressure micropipette manipulation results confirmed the range of adhesion force measured by the hydrodynamic flow of the 70 µm micropipette.

We applied the well-established step-pressure micropipette manipulation technique (SPT) [21]–[22] to validate the magnitude of the adhesion force calculated from the hydrodynamic simulations of the 70 µm micropipette. We measured a total number of *(n = 34)* monocyte cells originating from two different human donors. In these experiments, we applied a glass micropipette with an inner diameter of 5 µm instead of 70 µm to lift the cells with a diameter of ∼15 µm under hydrostatic conditions (Fig. 8). First we determined the height of cells in the culture by carefully approaching the cell with the micropipette and observing if the cell was disturbed. We found that the height of cells was in the 10–15 µm range. Then we positioned the tip above a new cell, adjusted the vacuum in the syringe, and opened the fluidic valve. To grab the cell we approached and touched it with the tip gently until the distance between the tip and the bottom of the Petri dish was decreased to 10 µm. Then we lifted the tip to 30 µm above the surface. If the cell was picked up we turned to the next cell. (Most cells were also sucked into the small micropipette after the detachment from the surface showing the cells’ capability of significant deformation.) If the cell remained on the surface we increased the vacuum. Suction force was increased in steps as long as the selected cell was removed. Adhesion force *(F_A_)* was calculated from the following formula:
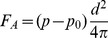
where *p_0_* and *p* are the hydrostatic pressure outside and inside the micropipette, respectively. *d* is the diameter of the micropipette. We calculated the ratio of adherent cells remaining on the surface after applying the next step of vacuum (Fig. 8). Step-pressure micropipette manipulation results confirmed the range of cell adhesion force calculated from the simulations of the flow induced by the 70 µm micropipette. To confirm hydrostatic conditions we completed flow measurements also in the 5 µm micropipette as described in the *Methods* section. In this case the flow rate was under the detection limit.

#### Correlation between the average cell area and cell adhesion strength

Whereas the average cell area of the macrophages and the monocytes was the largest and smallest, respectively (Figure S5 in File S1), dendritic cells and monocytes were the most and least adherent cells according to our measurements. We conclude that there is no obvious correlation between the cell area and adhesion force in case of these leukocyte cell types.

## Discussion

To gain statistically reliable information of cell adhesion, large numbers of cells have to be measured. Direct measurement of the adhesion force of single cells is challenging and extremely low throughput. We used a computer controlled micropipette mounted onto a normal inverted microscope for probing a large number of single cells interacting with specific macromolecules. The adhesion force of surface attached human monocytes and their descendants: macrophages and dendritic cells could be accurately probed by repeating the pick-up process with increasing vacuum. We estimated the hydrodynamic lifting force acting on single cells by numerical simulation of the flow in the micropipette. Populations of hundreds of cells adhered to specific macromolecules were measured one by one in the experiments. We blocked nonspecific cell adhesion by the protein non-adhesive PLL-g-PEG. We found that human monocytes are less adherent to fibrinogen than macrophages and dendritic cells, the latter producing the highest average adhesion force. The range of adhesion force we measured is comparable to the average force of 600 nN gained with the FluidFM (AFM combined with microfluidics) on HeLa cells [23], considering that this former result did not include highly adherent cells that were detached from the cantilever during lifting. Magnitude of the hydrodynamic lifting force was confirmed by the hydrostatic step-pressure micropipette manipulation technique. When slowly pulling the cells with the 5 µm micropipette cells showed significant deformation. Most of them were also sucked into the small micropipette as being detached from the surface. Vertical stretching of cells by the local force of the small micropipette is expected to break the adhesion bonds sequentially similarly to AFM [17] resulting in a lower overall force. We attribute the somewhat larger adhesion force measured with the 70 µm hydrodynamic micropipette to the homogenous vacuum acting simultaneously on the whole cell instead of the local pulling force of the 5 µm hydrostatic micropipette breaking molecular bonds sequentially. Microfluidic rectangular channel gave similar results for the relative adhesion strength of the 3 different cell types. It should be pointed out that the shear force needed to remove cells in the microfluidic channel was 2 orders of magnitude lower than the hydrodynamic lifting force measured by the micropipette. We attribute the difference to a presumed zipping effect, i.e., cells might be removed from the surface with a fraction of the total adhesion force if the shear stress serially breaks the molecular bonds starting from one end of the cell proceeding to the other end. Experiments on micro- and nanostructured surfaces also showed that the normal and lateral forces needed to detach a cell can have a different dependence on the texture of the surface [52]. Therefore, the micropipette adhesion test characterizes better the overall adhesion force of individual cells than the shear stress experiment. Moreover, using our technique, the probed single cells can be easily picked up and further investigated by other techniques; a definite advantage to exploit the computer controlled micropipette in state of the art biological research. A drawback of the technique as compared to AFM is its indirect nature. To calculate the value of the adhesion force hydrodynamic simulations have to be carried out. The calculated force acting on a cell can depend on the shape of the cell, e.g., when free-slip boundary condition is applied on the surface of the Petri dish. To ensure no-slip boundary condition we suggest to use cleaned glass substrate instead of plastic if the cell shape varies significantly. In the current study the cell shape of the 3 cell types was similar. Force spectroscopy performed with an AFM can show the process of detachment. To obtain kinetic data with the micropipette a high speed camera is needed to follow the removal of the cell in the flow.

Our experiments revealed the existence of a sub-population of highly adherent cells among macrophages and dendritic cells. We observed a strong effect of the shear flow on cell morphology in case of dendritic cells and macrophages. These cells became elongated and aligned to the direction of the flow in the microfluidic channel. Although this phenomenon has been described in case of endothelial cells [51] it is less documented for leukocytes. Similar effects were not observed in experiments with the micropipette, which infers that the interpretation of adhesion strength measurements is more straightforward when using the micropipette. Change of cell morphology is related with several other changes of the cytoskeleton affecting adhesion itself triggered by the shear stress unintentionally. We propose that this artefact can be eliminated when using the hydrodynamic lifting force of a micropipette instead of shear stress. Of course, when the aim of the experiment is to study the complex cellular response to a shear flow rather than measuring simply cell adhesion under static conditions, then a tangential flow may be necessary [51], [53].

As a next step the molecular background of the wide distribution of adhesion force in the same cell type can be explored using the automated micropipette combined with fluorescent labelling of cell surface adhesion proteins and single cell RNA sequencing.

## Supporting Information

File S1
**Figures S1–S5 and Table S1. Figure S1. Sketch of the experimental setup for measuring the flow rate in the micropipette as a function of vacuum (** ***p-p0*** **) in the syringe induced by increasing its volume to** ***V_1_*** **from the initial** ***V_0_*** **.** We also considered the effect of hydrostatic pressure due to *H_g_. Q*: flow rate in the micropipette. **Figure S2. CFD geometry showing the tip of the micropipette and the cell modelled with a hemisphere with a radius of** ***R*** **in the** ***φ = 10*** **degrees sector of space.** Wall thickness of the micropipette is *b*. Inner diameter of the micropipette opening is *D1. D2* defines the cone angle of the micropipette measured on the microscopic image of the glass micropipette (Fig. S3 in File S1). Distance between the bottom of the Petri dish and the tip of the micropipette is *H*: Boundary condition are as follows: OP – opening, OUT – outlet, NSW – no-slip wall, FSW – free-slip wall. SYM: side walls were set to symmetry. See also Table S1 in File S1. **Figure S3. Inner diameter of the glass micropipette we used in our experiments as a function of distance from its tip.** We determined the inner geometry of the micropipette on digital microscopic images after loading the tip with the solution of toluidine blue stain. **Figure S4. To analyze the effect of cell shape and positioning offset we ran 3D simulations.** Figure shows the hydrodynamic lifting force acting on the model cell with free-slip (a) and no-slip (b) boundary conditions at the bottom of the Petri dish. Computations were run with a hemisphere model of the cell with a radius of *R* in the center (axis of the micropipette) and also with the same model cell on the surface but pushed out of the center by 5 µm (eccentric position) to estimate the impact of the error of micropipette positioning on the lifting force. Positioning offset had negligible effect on the hydrodynamic lifting force. To analyze the effect of cell shape on the lifting force we used an oblate hemispheroid model cell with a major radius of *R* but minor radius (height) of *R/2, i.e.,* a twice as flat cell. The effect of cell shape was significant only in case of the free-slip boundary condition at the bottom of the Petri dish. All 3D computations were ran at 10 µm pipette height. **Figure S5. We examined the correlation between the average cell area and cell adhesion strength.** Monocytes (panel a, n = 709), macrophages (panel b, n = 2250), and dendritic cells (panel c, n = 2946) adhered onto the fibrinogen surface originating from two donors were recognized automatically in the large phase contrast mosaic images using the CellSorter software. On the basis of the width (Δ*x*) and height (Δ*y*) of the frames enclosing single cells we approximated the cell area *(A)* as follows: 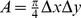. In the few cases when more than one cell were detected in the same frame, we excluded them from the calculation. Whereas the average cell area (panel d) of the macrophages and the monocytes was the largest and smallest, respectively, dendritic cells and monocytes were the most and least adherent cells, respectively, according to our measurements. We conclude that there is no obvious correlation between the cell area and adhesion force in case of these leukocyte cell types. **Table S1. Geometric parameters of the CFD model used in the numerical simulations.**
(DOCX)
