# Supplementary material for: Single Cell Adhesion Assay Using Computer Controlled Micropipette
Source: PLoS One. 2014 Oct 24;9(10):e111450. doi: 10.1371/journal.pone.0111450 (PMC4208850; doi:10.1371/journal.pone.0111450)
Supplement: File S1 — Figures S1–S5 and Table S1. Figure S1. Sketch of the experimental setup for measuring the flow rate in the micropipette as a function of vacuum ( p-p0 ) in the syringe induced by increasing its volume to V1 from the initial V0 . We also considered the effect of hydrostatic pressure due to Hg. Q: flow rate in the micropipette. Figure S2. CFD geometry showing the tip of the micropipette and the cell modelled with a hemisphere with a radius of R in the φ = 10 degrees sector of space. Wall thickness of the micropipette is b. Inner diameter of the micropipette opening is D1. D2 defines the cone angle of the micropipette measured on the microscopic image of the glass micropipette (Fig. S3 in File S1). Distance between the bottom of the Petri dish and the tip of the micropipette is H: Boundary condition are as follows: OP – opening, OUT – outlet, NSW – no-slip wall, FSW – free-slip wall. SYM: side walls were set to symmetry. See also Table S1 in File S1. Figure S3. Inner diameter of the glass micropipette we used in our experiments as a function of distance from its tip. We determined the inner geometry of the micropipette on digital microscopic images after loading the tip with the solution of toluidine blue stain. Figure S4. To analyze the effect of cell shape and positioning offset we ran 3D simulations. Figure shows the hydrodynamic lifting force acting on the model cell with free-slip (a) and no-slip (b) boundary conditions at the bottom of the Petri dish. Computations were run with a hemisphere model of the cell with a radius of R in the center (axis of the micropipette) and also with the same model cell on the surface but pushed out of the center by 5 µm (eccentric position) to estimate the impact of the error of micropipette positioning on the lifting force. Positioning offset had negligible effect on the hydrodynamic lifting force. To analyze the effect of cell shape on the lifting force we used an oblate hemispheroid model cell with a major radius of R but minor [file pone.0111450.s001.docx]

**Supporting Material**

**Single cell adhesion assay using computer controlled micropipette**

Rita Salánki ^1,2,3^, Csaba Hős ^4^, Norbert Orgovan ^2,3^, Beatrix Péter ^1,2^, Noémi Sándor ^5^, Zsuzsa Bajtay ^6^, Anna Erdei ^5,6^, Robert Horvath ^2^, Bálint Szabó ^2,3,7^ ^[[1]](#footnote-1)^


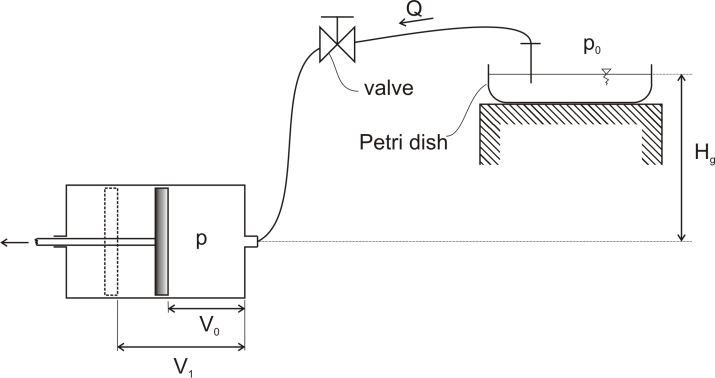


FIGURE S1 Sketch of the experimental setup for measuring the flow rate in the micropipette as a function of vacuum (*p-p0*) in the syringe induced by increasing its volume to *V_1_* from the initial *V_0_*. We also considered the effect of hydrostatic pressure due to *H_g_. Q*: flow rate in the micropipette.


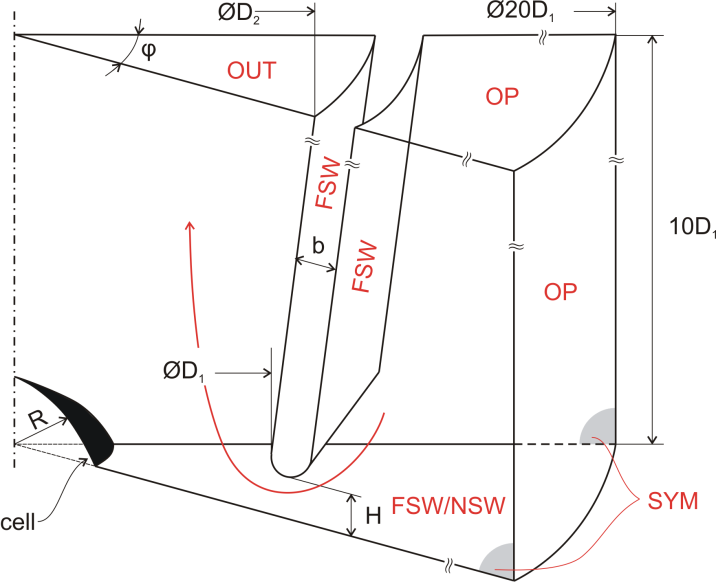


FIGURE S2 CFD geometry showing the tip of the micropipette and the cell modelled with a hemisphere with a radius of *R* in the *φ = 10* degrees sector of space. Wall thickness of the micropipette is *b*. Inner diameter of the micropipette opening is *D1. D2* defines the cone angle of the micropipette measured on the microscopic image of the glass micropipette (Fig S3). Distance between the bottom of the Petri dish and the tip of the micropipette is *H*: Boundary condition are as follows: OP – opening, OUT – outlet, NSW – no-slip wall, FSW – free-slip wall. SYM: side walls were set to symmetry. See also Table S1.

TABLE S1 Geometric parameters of the CFD model used in the numerical simulations.

| D_1_ [μm] | D_2_ [μm] | H [μm] | R [μm] | b [μm] |
| --- | --- | --- | --- | --- |
| 70 | 83 | 5, 10 | 10 | 25 |


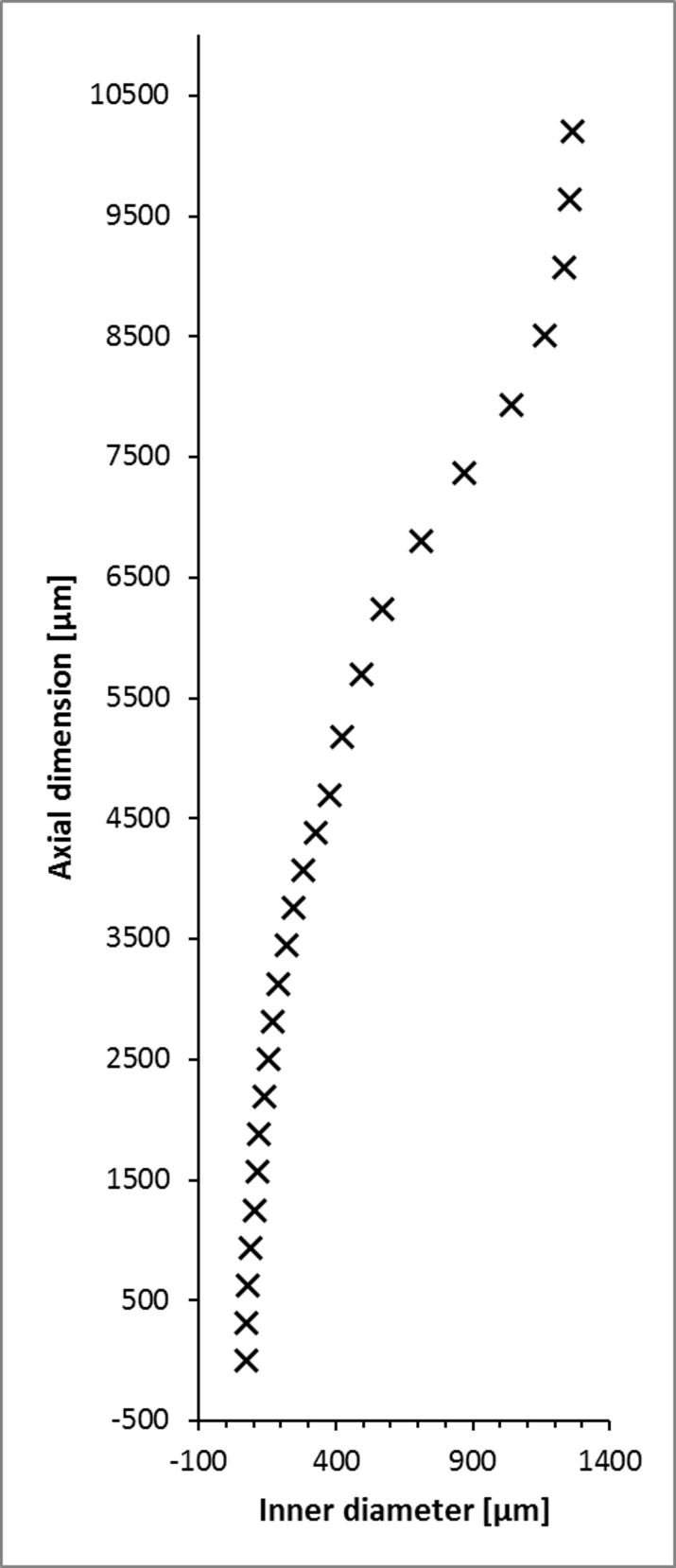


FIGURE S3 Inner diameter of the glass micropipette we used in our experiments as a function of distance from its tip. We determined the inner geometry of the micropipette on digital microscopic images after loading the tip with the solution of toluidine blue stain.


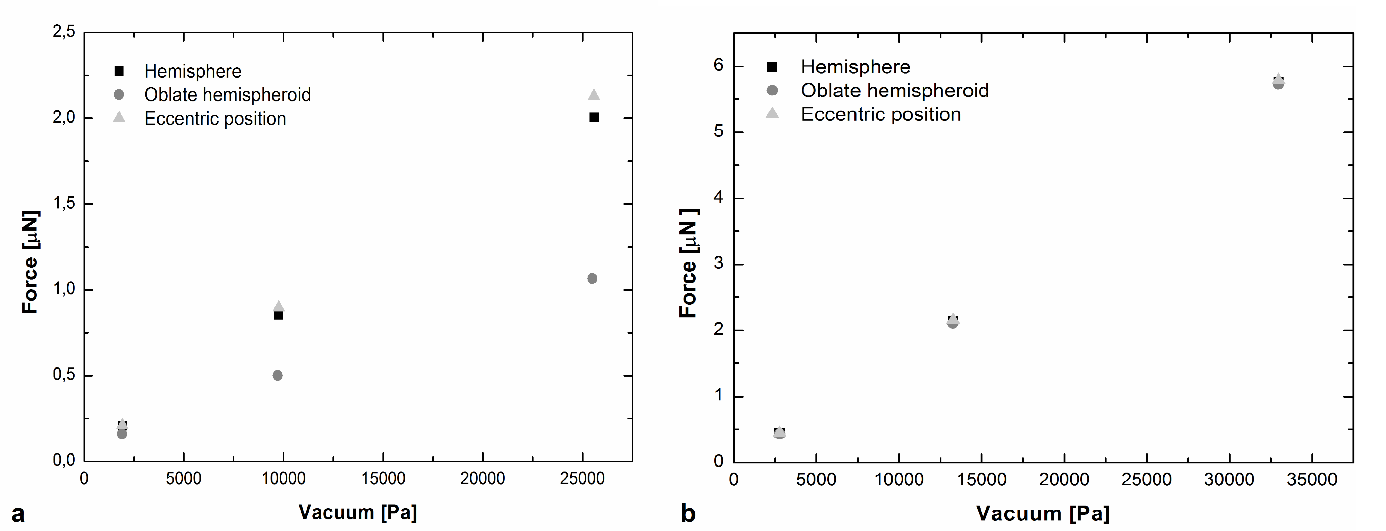


FIGURE S4 To analyze the effect of cell shape and positioning offset we ran 3D simulations. Figure shows the hydrodynamic lifting force acting on the model cell with free-slip (a) and no-slip (b) boundary conditions at the bottom of the Petri dish. Computations were run with a hemisphere model of the cell with a radius of *R* in the center (axis of the micropipette) and also with the same model cell on the surface but pushed out of the center by 5 µm (eccentric position) to estimate the impact of the error of micropipette positioning on the lifting force. Positioning offset had negligible effect on the hydrodynamic lifting force. To analyze the effect of cell shape on the lifting force we used an oblate hemispheroid model cell with a major radius of *R* but minor radius (height) of *R/2, i.e.,* a twice as flat cell. The effect of cell shape was significant only in case of the free-slip boundary condition at the bottom of the Petri dish. All 3D computations were ran at 10 µm pipette height.


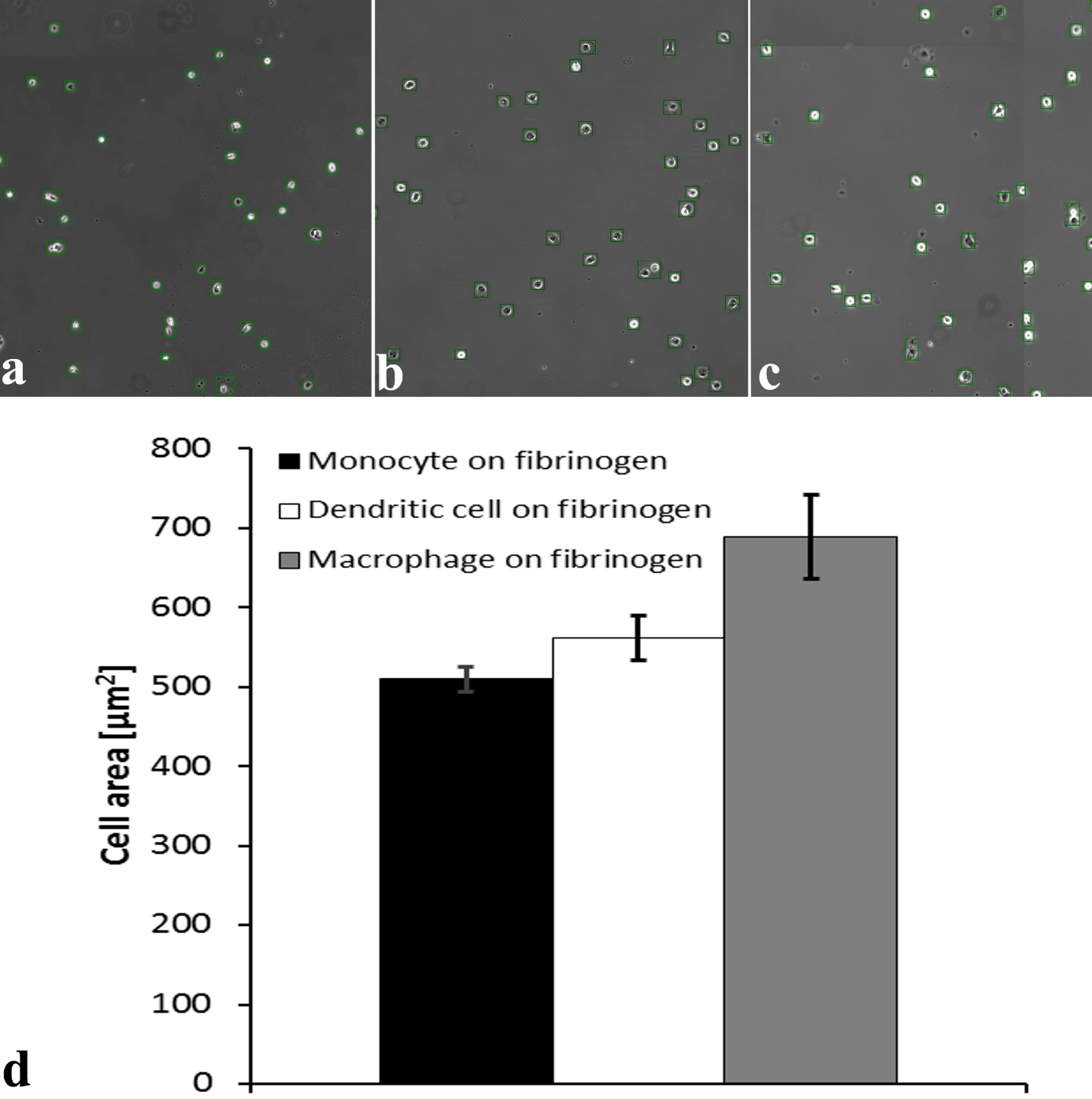


FIGURE S5. We examined the correlation between the average cell area and cell adhesion strength. Monocytes (panel a, n=709), macrophages (panel b, n=2250), and dendritic cells (panel c, n=2946) adhered onto the fibrinogen surface originating from two donors were recognized automatically in the large phase contrast mosaic images using the CellSorter software. On the basis of the width ($\Delta x$) and height ($\Delta y$) of the frames enclosing single cells we approximated the cell area *(A)* as follows:

$$A=\frac{\pi}{4}\Delta x\Delta y$$

In the few cases when more than one cell were detected in the same frame, we excluded them from the calculation. Whereas the average cell area (panel d) of the macrophages and the monocytes was the largest and smallest, respectively, dendritic cells and monocytes were the most and least adherent cells, respectively, according to our measurements. We conclude that there is no obvious correlation between the cell area and adhesion force in case of these leukocyte cell types.

1. To whom correspondence should be addressed. Email: [balintszabo1@gmail.com](mailto:balintszabo1@gmail.com) [↑](#footnote-ref-1)
